# Addressing the Disconnect between the Estimated, Reported, and True Rabies Data: The Development of a Regional African Rabies Bulletin

**DOI:** 10.3389/fvets.2017.00018

**Published:** 2017-02-20

**Authors:** Terence P. Scott, Andre Coetzer, Anna S. Fahrion, Louis H. Nel

**Affiliations:** ^1^Department of Microbiology and Plant Pathology, Faculty of Natural and Agricultural Sciences, University of Pretoria, Pretoria, South Africa; ^2^Global Alliance for Rabies Control, Manhattan, KS, USA; ^3^Department of Control of Neglected Tropical Diseases, World Health Organization, Geneva, Switzerland

**Keywords:** rabies, bulletin, DHIS2, data, surveillance, PARACON, reporting

## Abstract

It is evident that rabies continues to be a neglected tropical disease; however, a recent global drive aims to eliminate canine-mediated human rabies by 2030. Global efforts have been vested into creating and developing resources for countries to take ownership of and overcome the challenges that rabies poses. The disconnect between the numbers of rabies cases reported and the numbers estimated by prediction models is clear: the key to understanding the epidemiology and true burden of rabies lies within accurate and timely data; poor and discrepant data undermine its true burden and negate the advocacy efforts needed to curb this lethal disease. In an effort to address these challenges, the Pan-African Rabies Control Network is developing a regional rabies-specific disease surveillance bulletin based on the District Health Information System 2 platform—a web-based, open access health information platform. This bulletin provides a data repository from which specific key indicators, essential to any rabies intervention program, form the basis of data collection. The data are automatically analyzed, providing useful outputs for targeted intervention. Furthermore, in an effort to reduce reporting fatigue, the data submitted, under authority from the respective governments, can automatically be shared with approved international authorities. The implementation of a rabies-specific bulletin will facilitate targeted control efforts and provide measurements of success, while also acting as a basis for advocacy to raise the priority of this neglected disease.

## Introduction

Rabies has been a scourge for centuries. Despite the fact that this disease is fully preventable, the exemplary efforts that led to the elimination of dog rabies (e.g., in Western Europe, the United States, and Japan) have failed to be replicated across Asia and Africa. Significantly, the continued lack of impetus toward its control and elimination has resulted in the official recognition of rabies as a neglected tropical disease (1). The low priority status of rabies in the global community has resulted in a lack of interest from relevant stakeholders and national governments, despite the important socioeconomic and public health impact of the disease (2). Without a unified governmental and global effort toward rabies control, human lives will continue to be needlessly lost.

Many rabies endemic countries are afflicted by economic and political instability, resulting in a plethora of challenges. These circumstances make it even more difficult to assign sufficient resources toward rabies surveillance and intervention, especially when a host of other diseases competes for attention. Diseases are often prioritized according to those that are most visible or perceived to be emerging threats. Failure to command attention leads to a lack of resources required to implement an effective intervention strategy. A cycle of neglect is thus established, depriving the disease from the attention that it deserves (3). The absence of accurate data is considered as a foundational challenge in the prioritization of the fight against rabies (2, 4).

By providing decision makers, countries and regions with robust estimates as to both the burden of the disease (2) and the associated costs (5), a stronger case for rabies prioritization and intervention may be built. Therefore, an important next step would be for regional networks and the global community to improve data collection, quality, and analysis, among others, to inform the disease prioritization cycle.

## Disconnect Between Estimated and Reported Data

There is an inordinate disconnect between the data that are officially reported by governments and that of the estimates provided by predictive models (2). Furthermore, a plethora of data from grassroots levels (e.g., health-care facilities) is also available but is seldom reported and aggregated to the national level. It can be argued that these models provide the most useful and well-considered estimates for the burden of rabies to date, specifically in Africa and Asia where rabies reports, in those instances where reporting does occur, often appear to be haphazard or discrepant (6). Within Africa, for example, underreporting the actual numbers of human deaths can be as much as 100-fold, resulting in the misperception of the burden of rabies in individual countries and on the continent as a whole (7).

While a lack of reported data is problematic, poor-quality data can result in a conscious decision to mistakenly not address a disease. Poor-quality data can present a false picture of the situation, resulting in a false sense of security among governments and decision makers. Thus, improving data reporting should be a process that starts at the lowest governmental levels and aggregates up to the national level. The ultimate goal of each country and stakeholder involved in rabies control and elimination should be to drive accurate data collection, reporting, and analysis in order to ensure that the reported data reflect the true burden of rabies in their country.

## Hindrances to Reliable Data Collection

Many countries in Africa have functional surveillance systems, yet the data that are reported at a national level or to international organizations such as the World Health Organization (WHO) and World Organization for Animal Health (OIE) do not correlate with that from the community or district level (6, 8–11) as it is sourced from various sectors and databases within the country.

Cumbersome, complex, and time-consuming reporting systems contribute in large to poor data collection and reporting. A recent study showed that only 10/19 surveyed African countries reported human rabies data using an electronic database, but 18/19 countries still used paper-based reporting at some stage(s) within their reporting system (12). Rabies data reports are often late, or submission rates are low. A stand-alone rabies surveillance system was reported in 4/16 countries, meaning that reported data for the remaining 12 countries would likely be presented to an integrated disease surveillance system for multiple diseases within each ministry (12). As a result of the complex structuring of existing types of reporting systems, as well as the lack of any feedback mechanisms, those data misreported or unreported are not pursued, clarified, or questioned (9). These factors all contribute to a lack of confidence in rabies surveillance systems in Africa; only 3/19 countries deemed their rabies surveillance system adequate, despite its status as a notifiable disease in all 19 surveyed countries (12).

Apart from the obvious drawbacks associated with cumbersome reporting systems, the roles played by data managers should also be considered. Individuals responsible for reporting data are tasked with various other responsibilities. These include reporting morbidity and mortality statistics to international organizations and responsibility for reporting data for all of the notifiable diseases to various sectors, stakeholders and decision makers within a country. Additionally, there should be capacity for analysis and interpretation of raw data in order for it to be used for case follow-up, outbreak responses, targeted intervention campaigns, and the identification of disease incidence hotspots. Thus, data managers are often overburdened with a plethora of tasks and commitments, resulting in some health data being poorly reported. In this light, it would be most beneficial for data reporting as a whole if reporting systems could be made as simple and efficient as possible.

## Improving Data Reporting in Africa

Formerly, two main international epidemiological reporting bulletins for rabies existed, namely the WHO Rabnet (13)—reporting on public health data—and the OIE World Animal Health Information System (WAHIS) (14)—reporting on veterinary health data. The Rabnet system closed in 2011 due to a lack of reported data (8). The WAHIS is a well-established global animal disease reporting system that reproduces the data that countries submit to OIE, but it is also limited by the underreporting issues inherent to the national reporting systems (7). A further disconnect exists in many places between human and animal health, with little or no cross-sectoral exchange occurring (8). Thus, the need for regional, One Health-oriented reporting networks has become evident (15). The creation of rabies-specific regional bulletins has been exceptionally successful in the Pan-American Health Organization region (SIEPI database) (16) and in Europe (WHO Rabies Bulletin Europe) (17). These bulletins have enabled countries to improve surveillance for targeted intervention and control measures, while also increasing advocacy and awareness about the disease situation. In Africa, no rabies-specific database existed, and only informal reporting occurred within regional networks (6, 12). Also, with the lack of a regional bulletin, data quality from the African region has remained poor or non-existent, emphasizing the need for such a tool.

### Addressing the Disconnect in Africa: The Pan-African Rabies Control Network (PARACON) Bulletin

The PARACON was founded in 2014 under the secretariat of the Global Alliance for Rabies Control. PARACON unified various subregional networks and other independent countries into a single regional network for Africa, with the mandate to provide tools and support to Member countries (15). In light of this mandate, PARACON is developing and refining a regional, network-based reporting bulletin for rabies using a standardized set of critical indicators that are applicable to each country individually, as well as to the region as a whole. Furthermore, to improve reporting incrementally on a global scale, WHO is working closely with regional networks to improve rabies data collection (8). Thus, through the unification of rabies reporting systems in the region, and the creation of a single platform that can provide regional rabies data to global reporting systems, the burden of data reporting can be substantially reduced.

#### District Health Information System 2 (DHIS2): The Backbone of the PARACON Bulletin

The DHIS2 is an open-source platform specifically designed for health surveillance. It functions not only as a data collection and storage point but also as a versatile tool to aggregate and disaggregate data, manage individual patient records, perform analytics, and create useful and interactive visuals for data analysis. In addition, to facilitate the use and dissemination of the data, the DHIS2 system has an integral sharing functionality with mass communication and messaging capabilities, while maintaining strict access control and confidentiality. The DHIS2 can furthermore be adapted to any type of program requiring data gathering, analysis, and dissemination.

The DHIS2 has been implemented globally, including in 40 countries throughout Africa (18, 19) and is currently being used as an official Health Information System (HIS) in Malawi, Kenya, and Uganda, among others (19). In Africa, for instance, the implementation of the DHIS2 system in Zimbabwe resulted in timelier reporting, enhanced data quality, and improved report completeness for child health-related data (20). Similarly in Zambia, the DHIS2 system was used for a national malaria control program, resulting in improved case detection, improved diagnostic confirmation, and reduced numbers of unconfirmed malaria cases reported (21).

#### The PARACON Bulletin

As a new way of rabies data collection and dissemination, the PARACON bulletin uses the DHIS2 as its data collection platform. The PARACON bulletin will encourage the collection of rabies data from both the public and animal health sectors, promoting the One Health concept and intersectoral collaboration for improved rabies surveillance efforts. It is hoped that the simplicity of the Bulletin’s use and design will lead to improved rabies data reporting with little input and expertise required. Initial feedback at the Beta launch of the PARACON bulletin in 2016 was positive: 65% (15/23) of West African PARACON workshop participants agreed that the bulletin is easily workable and could be utilized as a country-level solution. Subsequent to its launch in July 2016, the PARACON bulletin has been implemented at another workshop targeting five Anglophone PARACON countries, where all of the participants agreed that the PARACON bulletin is as an easy-to-use and useful rabies reporting system for their country. In addition, the PARACON bulletin will continue to be introduced to the remaining PARACON member countries in subsequent workshops, meetings and in-country visits. Outcomes from these workshops and visits have already shown continued data reporting for the end of 2016, with continuous follow-up for further data reporting.

The use of standardized data indicators is paramount to the consistent and accurate reporting of data for national, subregional, regional, and international uses. By initially focusing on basic indicators (Table 1) during the early phases of implementation, the Bulletin is designed to be easy to use and should provide the maximum output in exchange for a limited input. This will be essential to encourage countries to buy-in to the concept. The initial indicators will likely adapt as the bulletin continues to develop in line with the global needs and trends in data collection for rabies and are also subject to further consultation and refinement. As PARACON will work closely with WHO as well as other international collaborating centers and expert networks, the concept is to create a globally accepted, standardized set of indicators that will continue to be developed and refined. This standardized set of indicators will be focused on essential criteria to help guide countries in (further) developing their own surveillance and control strategies as they strive toward self-declaration of freedom from rabies.

**Table 1:** **Description of and rationale for using initial basic indicators in the Pan-African Rabies Control Network bulletin**.

Indicator	Disaggregation	Description	Rationale	Reporting period
Number of bite cases in humans	Age: <5 years, 5–14 years; ≥15 years; unknown age Sex: male, female; unknown Wound category: I, II, or III	Number of bite cases reported at a health-care facility, disaggregated by age, sex, and wound category	To determine at-risk populations (children, adults) and the numbers of people who have been potentially exposed to a rabid animal; this indicator influences decisions regarding human vaccine procurement and targeted education. This indicator also excludes snake bites	Annual

Doses of human vaccines purchased	None	Number of human vaccines purchased for the country	To determine the number of vaccines available in the country and whether this complies with PEP requirements	Annual

Cost per vaccine (US$)	Private sector Public sector	Cost per vaccine administered in a government institution (including all associated costs such as doctor’s fees, consumables, etc.)	To determine the costs associated with procurement and administration of vaccine for budgetary purposes and to advocate the allocation of funds toward rabies control efforts	Annual

Doses of animal vaccine available	Purchased this year Viable vaccine carried over from previous year Vaccine administered	Number of animal vaccines administered, carried over, and purchased by the government for mass vaccination campaigns	To establish the number of vaccine doses available to the government; this indicator is also used for the eventual calculation of the estimated vaccination coverage for the country	Annual

Estimated total dog population	Human population: urban, rural Human:dog ratio: urban, rural Dog population: urban, rural	A means to determine an estimated dog population for the country based on the HDR method (4, 22–24)	In most countries where rabies is endemic, there is no information about their current dog population; this lack of knowledge inhibits the assessment of the effectiveness of mass dog vaccination campaigns and also prevents countries from purchasing the correct number of doses of animal vaccine to achieve 70% coverage	Annual

Dog vaccination coverage		A means to determine the estimated vaccination coverage for the estimated dog population for the country	To enable decision makers to determine whether sufficient vaccine has been purchased and administered and for countries to plan ahead for vaccine purchase for the next year	Annual

Animal rabies cases	Species: dog, cat, livestock, wildlife, bat Result: positive, negative Total: per species, per result	Determination of the number of suspect rabies cases submitted for laboratory confirmation. The results indicate the number of positive and negative cases per species, as well as the totals	Results to provide an indication of the effectiveness of a surveillance program by examining the positive:negative ratio	Biannual

Human rabies cases	Diagnosis: clinical, laboratory Result: positive, negative	The number of human rabies cases diagnosed clinically and by laboratory confirmation	To determine the burden of the disease and to determine the efficacy of disease intervention strategies	Biannual

Number of people receiving PEP	Sex: male; female; unknown Age: <5 years, 5–14 years; ≥15 years; unknown age Wound category: I, II, or III	Number of humans receiving wound care and at least one dose of rabies vaccine for PEP	To determine the number of people receiving at least one dose of PEP in a country	Annual

*HDR, human:dog ratio; PEP, post-exposure prophylaxis*.

On a regional scale, the use of a single reporting system can instigate interest and control efforts in neighboring countries through the evaluation of shared data, in the race for the first African country to declare freedom from canine-mediated human rabies by 2030. Furthermore, by sharing data internationally, countries can work together to control rabies cycles along international borders—something that is vitally important for a sustainable approach to rabies control (3). As the Bulletin is closely linked to the Stepwise Approach towards Rabies Elimination tool, as well as to key indicators for the WHO Global Health Observatory, this platform presents an easy means for countries to assess progress and program successes, while gathering essential data for the declaration of freedom from rabies in the future.

The PARACON bulletin has been designed to not only act as a regional platform for national rabies data but also can be adopted by countries as their own national and subnational rabies surveillance systems. The use of an electronic platform will enable users to access data instantly, resulting in an improved sense of ownership (25). To ensure that data quality is kept at the highest possible standards, several data quality checks will be available, including completeness, correctness, and timeliness of reporting. Reported data will thus remain of the utmost quality, ensuring its usefulness to decision makers and stakeholders. Customized reports can be created and tailored to each sector’s needs—with only selected indicators included into each report—making them relevant to that specific sector or authority. Additionally, the PARACON Bulletin enables automatic analyses and visuals that can help target intervention programs toward high-risk areas in a country.

Often, donor-based funding projects that aim to improve surveillance initiate similar reporting bulletins, but they end abruptly if those donors discontinue support and maintenance and administration are not sustained (26). A bulletin hosted and administered by an independent organization that has no direct involvement in target countries, as is the case with the PARACON bulletin, would ensure the sustainability of programmes. The PARACON network enables countries to start small, making the initial steps toward improving surveillance a less daunting task. Furthermore, direct international support will allow subregional focal points, WHO country offices and other subregional or national authorities to assist countries and contribute valuable data to this regional bulletin.

Although the PARACON bulletin will help to improve the surveillance networks of individual countries, it cannot compensate for a system lacking any basic surveillance. The first step toward collecting reliable and realistic data reflecting the true rabies situation is awareness at the community level and the subsequent collection of relevant data. While in some countries, surveillance is hampered because no laboratory confirmation exists, a lack of surveillance can generally be attributed to the logistical constraints associated with sample submission (27, 28). Without a surveillance foundation, any data collection system will be a redundant tool. Thus, countries will need to implement a collective plan for improving rabies surveillance concurrently with the implementation of the PARACON bulletin. What needs to be known and what minimum requirements are to be met to establish adequate rabies surveillance for both humans and animals has recently been compiled in the rabies surveillance blueprint (29). In doing so, the PARACON bulletin will build upon these foundational elements and can also be integrated into existing HIS to improve data flow.

## Conclusion

The lack of awareness and knowledge about the true burden and impact of rabies within countries in mainland Africa remain the primary barrier to the control and elimination of this disease. With poor rabies surveillance throughout Africa, there remains little political will and motivation to prioritize rabies among other notifiable diseases, resulting in its continued neglect. Improved surveillance is likely to lead to increased interest and more targeted and sustainable control strategies.

Pan-African Rabies Control Network has committed to provide countries with an effective, simple, and free-to-use bulletin for rabies that aims to address the issues of poor reporting and data quality in Africa as a whole. With the successes of other regional rabies reporting bulletins, as well as the addition of the PARACON bulletin for Africa, the next step would be to extend these regional databases into a global collective. With the unification of national data reporting systems and the reduced need for redundant reporting and the use of streamlined and automated data management systems, the expansion of regional bulletins into a global rabies-specific disease database is practicable. As a result, reporting burdens will be relieved and with improved data, decision makers and stakeholders can be convinced that rabies elimination is feasible within their country. With these tools and the support from the international community, the recent global declaration for the elimination of canine-mediated human rabies by 2030 (30) seems a realistic target for countries to aspire toward.

## Author Contributions

All of the authors contributed equally to the conception, development and drafting of the manuscript.

## Conflict of Interest Statement

The authors declare that the research was conducted in the absence of any commercial or financial relationships that could be construed as a potential conflict of interest.
